# Modeling and simulation of heat and mass transfer in an Ethiopian fresh *injera* drying process

**DOI:** 10.1016/j.heliyon.2021.e06201

**Published:** 2021-02-11

**Authors:** Alamrew B. Solomon, Solomon W. Fanta, Mulugeta A. Delele, Maarten Vanierschot

**Affiliations:** a Department of Chemical Engineering, Kombolcha Institute of Technology, Wollo University, Ethiopia; b Faculty of Chemical and Food Engineering, Bahirdar Institute of Technology, Bahirdar University, Ethiopia; c KU Leuven, Department of Mechanical Engineering, B-3000 Leuven, Belgium

**Keywords:** Diffusion, Drying, Injera, Mathematical modeling, Simulation

## Abstract

In this paper, we developed a mathematical model to simulate the heat and mass transfer during the convective drying of *injera*. The coupled set of heat and moisture partial differential equations (PDEs) were numerically solved by the finite element method (FEM) using COMSOL Multi-physics, 5.5. To validate the simulated results, drying experiments were performed using a tunnel dryer at two air temperatures (313.15 and 333.15 K) and velocities (0.25 and 0.5 ms^−1^). The predicted versus the experimental results showed a very good agreement with a coefficient of determination, $R^{2}>0.95$ for both temperature and moisture ratio and a Root Mean Square Error, RMSE < 0.05 for moisture ratio and <3.5 K for temperature. The predicted temperature and moisture ratio distributions of the *injera* at different times and positions (thickness and diameter) clearly showed the uniformity of drying. The time required to reduce the moisture ratio of *injera* from 1 (-) to 0.03 (-) at a temperature of 333.15 K, relative humidity of 11% and air velocity of 0.5 ms^−1^ was 125 min. Both temperature and velocity have a significant effect on moisture reduction when drying was conducted (p < 0.05). The interaction effect between them also indicates a significant difference (p < 0.05) in the moisture removal rate of *injera*.

## Nomenclature

**Abbreviation**
**AOAC**: Association of Official Analytical Chemists
**CHMC**: Convective Heat and Mass Coefficients
**FAO**: Food and Agricultural Organization
**FEM**: Finite Element Method
**HAD**: Hot Air Drying
**MR**: Dimensionless Moisture Ratio -

**Greek symbols**
$\beta_{1},\beta_{2},\beta_{3}$: Drying kinetics constants -
$\kappa_{1},\kappa_{2},\kappa_{3},\kappa_{4}$: Drying kinetics constants -
ϵ: Porosity of sample
ρ: Density of sample kg/m^3^
$\alpha ,\alpha_{2}$: Drying kinetics constants -

**Roman symbols**
**c**: Concentration mol/m^3^
**C**: Molar density mol/m^3^
Cp: Specific heat capacity J/kg.K
$D_{wa}$: Water air diffusivity m^2^/s
Dva: Vapour air diffusivity m^2^/s
Ei: Volume fraction of components -
**K**: Thermal conductivity W/m.K
$K_{ip}$: Intrinsic permeability -
**m**: Mass of the sample g
$m_{s}$: Mass of the sample after ashing g
$m_{a}$: Mass of the sample before ashing g
**p**: Pressure Pa
**R**: Universal gas constant J/molK
**r**: Personal correlation coefficient
**RH**: Relative humidity %
Sw: Saturation of water -
Sh: Heat source W/m^3^
Sm: Mass sources mol/(m^3^.s)
T: Temperature K
**u**: Velocity ms^−1^
**x,y,z**: Coordinates m

**Subscripts**
∂: Partial
∇: Radiant
a: Air
**eff**: Effective
**evap**: Evaporation
**i**: Component of the sample
**in**: Solid (injera)
**l**: Liquid
**sat**: Saturation
**v**: Vapor
**w**: Water

## 1 Introduction

*Injera* is one of the leading staple foods and is widely consumed in Ethiopia and some areas of Eritrea, Somalia and Sudan (Tesfay et al., 2014). It is a flat, round, pancake like product prepared from the flour of the super grain teff [Eragrostistef (Zucc.) Trotter], sorghum, barely or combinations of those (Satheesh and Fanta, 2018). Teff is an indigenous tropical cereal to Ethiopia (Neela and Fanta, 2020). At first, it was not so much appreciated until researchers discovered that teff contains no gluten, which makes it very appealing as healthy nutrition (Gebremariam et al., 2014). In recent years, special attention has been given to the benefits of *injera* and many types of research have studied teff-based food products (Gebremariam et al., 2015). Despite this fact, *injera* is not shelf stable and gets spoiled after three or four days of storage at surrounding temperature due to spoilage molds (Ashagrie and Abate, 2012). Apparently, food materials who have been dried in an open field using sun drying to control spoilage, are not suitable for large scale production due to inconsistent ambient conditions and possibilities of contamination with dust and insect infestations or spoilage related to moisture re-absorption (Ertekin and Yaldiz, 2004). Nowadays, scientifically suggested convective drying technologies are devised as an important tool to preserve food products (Tripathy and Kumar, 2008). Convective air flow around the food product has two main purposes: one is the transfer of heat to evaporate the moisture within the food and the second one is to remove the formed vapour. As such, the reduction in moist content promotes food preservation by avoiding microbial growth or reducing chemical reactions, both causing deterioration of the food product (Law et al., 2016; Vásquez-Parra et al., 2013). The modeling of simultaneously occurring heat and mass transport phenomena involved in food drying is complicated as it involves several interrelated physical phenomena in the evaporation process (Datta, 2007b). As such, mathematical tools like COMSOL Multiphysics, MATLAB and ANSYS are imperative to solve the heat, mass and momentum transport equations, including the porous media modeling of food materials (Datta, 2007a). An interesting numerical model to solve those coupled equations is computational fluid dynamics (CFD) as it can simultaneously solve the fluid flow in combination with heat and mass transfer. (Aregawi et al., 2014a; Datta, 2007b). Post-processing the numerical data can help in describing the involved physical phenomena, which can then afterwards be optimised to enhance the overall drying process and product quality (Bennamoun et al., 2015; Bird et al., 2015). The most critical step in the numerical model development is the choice of material property values. Biological materials are often anisotropic in behavior and it is not always possible to neglect this heterogeneity and apply bulk conditions. Also, several properties, like for instance density, thermal conductivity, moisture diffusivity etc., are dependent on temperature, composition, humidity and time during the drying process (Berk, 2018; Datta, 2007a; Feyissa et al., 2013). This variation needs to be incorporated in various simulations of heat and mass transport phenomena used in the design of food matrix storage and drying processes. Hence mathematical models need to be developed which take these variations into account in order to have accurate simulations (Datta, 2007b). Given the importance of the above-mentioned, the goal of the study in this paper is to investigate the applicability of several mathematical models to simulate heat and mass transport phenomena during the drying process of *injera*. The model results were experimentally validated by comparing the mean moisture content. Finally, the influence of process temperature on the moisture removal rate was evaluated and the time required to obtain the desired moisture content was determined.

## 2 Mathematical model development

Mathematical modeling is a good tool to obtain solutions for optimising drying related problems. It is well suited for evaluating the effect of various process parameters (temperature, velocity and humidity) and drying time (Kumar et al., 2014). Developing a drying model for food products including the relevant physical phenomena is a very challenging task. The main reason is the complex structure of food products and the change of their material properties during the drying process. Moreover, heat and mass transport are both highly coupled during drying, making it a very complex process. As such, modeling assumptions are indispensable, but these should be carefully made to ensure sufficient representation of the involved physics (Datta, 2007b).

### 2.1 Governing equations

Steady-state momentum and unsteady heat and mass transfer modeling during Ethiopian fresh injera drying was developed based on Newton's, Fourier's and Fick's laws. The model applied for momentum, continuity, mass and heat transfer with representative governing equations is described in Eqs. (1), (2), (3) and (15) respectively.

#### 2.1.1 Momentum equations

Based on the conservation of momentum and continuity, the governing equations for stationary air flow around the product to dry are given by

(1) $$\rho (u\cdot \nabla u)=-\nabla p+\nabla \cdot [\mu (\nabla u+{\left(\nabla u\right)}^{T})],$$

(2) ∇⋅u=0,

where *ρ* is the density (kg/m^3^), *μ* is the dynamic viscosity (Pa.s), u is the velocity vector (ms^−1^) and p is the pressure (Pa) (Bird et al., 2001, Bird et al., 2015).

#### 2.1.2 Mass transfer equation

Fick's Laws of diffusion can be formulated in terms of the material flux, which is the rate of substance transported per unit area (Bird et al., 2015).

(3) $$\underset{\text{I}}{\underset{︸}{\frac{\partial c}{\partial t}}}+\underset{\text{II}}{\underset{︸}{\nabla \cdot (-D\nabla c)}}+\underset{\text{III}}{\underset{︸}{u_{i}\cdot \nabla c}}=\underset{\text{IV}}{\underset{︸}{S_{m}}}$$

The first, second and third term on the left side of Eq. (3) denotes the accumulation of moisture, moisture transport due to diffusion and due to convection respectively. The right side term ($S_{m}$) is the evaporation mass flux. The moisture concentration (c) is related to the wet base moisture content according to (Law et al., 2016),

(4) $$c=\frac{M_{wb}\;\rho_{p}}{M_{w}},$$

where $M_{bw}$ is the moisture content (kg/kg wet base), $\rho_{p}$ is the sample density (kg/m^3^) and $M_{w}$ is the molecular mass of water (kg/mol), D in Eq. (3) is the diffusion coefficient and is defined as (Shen et al., 2020),

(5) $$D=D_{va}\epsilon^{3/4}S_{g}^{10/3},$$

where $D_{va}$ represent vapor-air diffusivity and $S_{g}$ gas saturation. The velocity field ($u_{i}$) was obtained from Darcy's law (Datta, 2007a) and defined as

(6) $$u_{i}=\frac{k_{i}}{\mu }\Delta p,$$

(7) $$k_{i}=k_{ii}k_{ir},$$

where Δ*p* is the pressure gradient, $k_{i}$ the permeability of the fluid as shown in Eq. (7), $k_{ii}$ represents the intrinsic permeability of water or gas at fully saturated state and $k_{ir}$ represents the relative permeability of water ($k_{wr}$) or gas ($k_{gr}$) with phase variations between 0 no phase) and 1 (saturated phase) in food materials. Measuring the intrinsic permeability value of food material is difficult and some reasonable approximation can be made by

(8) $$k_{wr}=\left\{ \begin{array}{c} {\left(\frac{S_{w}-S_{ir}}{1-S_{ir}}\right)}^{3}\;\text{for}\;S_{w}>\frac{1}{1.1}\\ 0\;\text{for}\;S_{w}<\frac{1}{1.1} \end{array},\right.$$

(9) $$k_{gr}=\left\{ \begin{array}{c} 1-1.1S_{w}\;\text{for}\;S_{w}<\frac{1}{1.1}\\ 0\;\text{for}\;S_{w}>\frac{1}{1.1} \end{array},\right.$$

where $S_{w}$ is the water saturation and defined as $\frac{M_{w}c}{\epsilon \rho_{w}}$, $S_{ir}$ is the irreducible liquid saturation expressed as $2.88(\frac{1}{T_{t=0}}\ln{\left(\frac{1}{\epsilon }\right)}^{0.48})$ according to (Datta, 2007b). The source term $S_{m}$ in Eq. (3) incorporates the change of phase between liquid water and vapour (Kuznetsov and Nield, 2013) and is defined through the following expression

(10) $$S_{m}=K_{vap}(a_{w}c_{v,sat}-c),$$

where $S_{m}$ is the mass source flux due to evaporation from the sample surface in (mol/m^3^.s), $K_{vap}$ is an evaporative constant considering strong evaporation (s^−1^), $a_{w}$ is the water activity which is a function of the dry base moisture content ($X_{db}$), Eq. (11) (Datta, 2007a), $c_{v,sat}$ is the concentration of saturation and is related to the saturation pressure Eq. (13) $p_{v,sat}$ which is a function of temperature.

(11) $$a_{w}=\exp(-0.0267X_{db}^{-1.656}+0.0107X_{db}^{1.51}\exp(-1.287X_{db})),$$

(12) $$X_{db}=\frac{c_{l}+c_{v}}{(1-\epsilon )\rho_{p}},$$

(13) $$C_{v,sat}=\frac{p_{v,sat}}{RT},$$

(14) $$p_{v,sat}=\exp(-5800.2206T^{-1}+1.3915-0.0486T+4.176x10^{-5}T^{2}-1.445x10^{-8}T^{3}+6.546\ln(T)),$$

where $X_{db}$ is the moisture concentration in dry base which is the ratio of fluid present in a sample to dry solid matrix and R is the universal gas constant.

#### 2.1.3 Heat transfer equation

The law of thermal conduction flux in a solid matrix was first formulated by Jean-Baptiste Joseph Fourier (Bird et al., 2015) and the general governing equation for heat transport including convection can be given by (Bird, 2004; Bird et al., 2015),

(15) $$\underset{\text{I}}{\underset{︸}{{\left(\rho C_{p}\right)}_{eff}\frac{\partial T}{\partial t}}}+\underset{\text{II}}{\underset{︸}{\nabla \cdot (-k_{eff}\nabla T)}}+\underset{\text{III}}{\underset{︸}{(\rho_{f})(C_{pf})u_{i}\cdot \nabla T}}=\underset{\text{IV}}{\underset{︸}{S_{h}}}.$$

The first, second, third and fourth term of Eq. (15) denotes the accumulation of heat, conduction, convective and source term respectively. The term ${\left(\rho Cp\right)}_{eff}$ is the effective volumetric heat capacity, $k_{eff}$ is the effective thermal conductivity, $\rho_{f}$ is the density of the fluid and $Cp_{f}$ is the thermal conductivity, which are defined as following:

(16) $${\left(\rho Cp\right)}_{eff}=\rho_{p}Cp_{p}-(1-\epsilon )\rho_{f}cp_{f},$$

(17) $$k_{eff}=\epsilon k_{p}+(1-\epsilon )k_{f},$$

(18) $$k_{f}=k_{w}-(k_{w}-k_{a})X_{v},$$

(19) $$\rho_{f}=\rho_{w}-(\rho_{w}-\rho_{a})X_{v},$$

(20) $$Cp_{f}=Cp_{w}-(Cp_{w}-Cp_{a})X_{v}.$$

The porosity of *injera* (*ϵ*) in Eqs. (16) and (17) is defined as

(21) $$\epsilon =1-\frac{\rho_{s}}{\rho_{b}},$$

where $\rho_{s}$ is the density of the solid and $\rho_{b}$ is the bulk solid including air and water (Datta, 2007b). The thermophysical properties in Eqs. (16) and (17), density (*ρ*), thermal conductivity ($k_{p}$) and specific heat capacity ($C_{p}$) are important for modeling and simulation of processes involving heating and cooling (Datta, 2007a) and are calculated by Eqs. (22) to (24) respectively,

(22) $$\rho =\frac{1}{\sum \frac{x_{i}}{\rho_{i}}},$$

(23) $$C_{p}=\sum_{i=1}^{n}(c_{pi}X_{i}),$$

(24) $$k=\sum_{i=1}^{n}(k_{i}E_{i}),$$

(25) $$E_{i}=\frac{x_{i}/\rho_{i}}{\sum x_{i}/\rho_{i}.}$$

The index i in Eqs. (22) to (24) represents the components of *injera* (protein, fat, ash, carbohydrate and water). The source term ($S_{h}$) from the air interface towards the sample in Eq. (15) is the product of latent heat of evaporation and mass source which is defined as

(26) $$S_{h}=H_{evap}M_{v}S_{m},$$

where $S_{h}$ is heat source (W/m^3^), $H_{evap}$ is the latent heat of evaporation (J/kg) and $M_{v}$ is the molecular weight of vapor (kg/mol).

### 2.2 Computational domain and initial and boundary conditions

**Figure 1:**
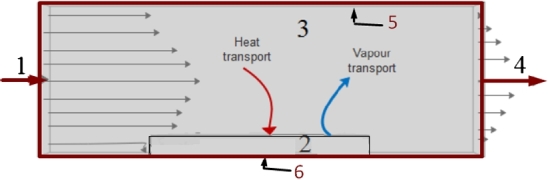
2D schematic diagram of the drying equipment (1) inlet, (2) Injera sample, (3) air zone, (4) exit, (5) top wall, (6) bottom wall.

The computational domain and the boundary and initial conditions of *injera* are illustrated in Fig. 1. At the inlet (1), where dry hot air enters the channel in the *x*-direction, uniform profiles of velocity and temperature are imposed, together with zero vapour concentration ($u=u_{t=0}$, $T=T_{t=0}$, $c_{v}=0$). Fully developed flow conditions are used at the outlet (4) of the channel ($\frac{\partial c}{\partial n}$=$\frac{\partial T}{\partial n}=0$, with *n* the normal direction to the boundary). No-slip conditions with adiabatic walls for top (5) and bottom (6) are utilized (u=v=w=0, $\frac{\partial T}{\partial n}=\frac{\partial c}{\partial n}=0$). Initial temperature and concentration in the sample domain are 298.15 K and 49,750 mol/m^3^ respectively.

#### 2.2.1 Initial condition

The initials values, constants and thermophysical properties of the *injera* sample are given in Table 1, Table 2.

**Table 1:** Initial and constant input parameters.

Symbol	Value (units)	Reference
*p*_*t*=0_	1 *atm*	Current study
*c*_*t*=0_	$\frac{S_{l}\rho_{w}\epsilon }{M_{w}}$ = 49,750 mol/m^3^	Current study
*T*_*t*=0_	323.15 & 333.15 K	Current study
*U*_*t*=0_	0.25 & 0.5 ms^−1^	Current study
*D*_*wa*_	2.6e-5 m^2^/s	(Cengel and Ghajar, 2011)
*D*_*va*_	2.6e-5 m^2^/s	(Cengel and Ghajar, 2011)
*H*_*evap*_	2.454e+6 J/kg	(Kumar et al., 2014)
*ϵ*	0.74	Current study

**Table 2:** Thermophysical properties of *injera*, air and water.

Symbol	Value (units)	Reference
	Density	
*ρ*_*in*_	*ρ*(*x*,*T*) (kg/m^3^)	Eq. (22) Current study
*ρ*_*a*_	1.25 kg/m^3^	(Karim and Hawlader, 2005)
*ρ*_*w*_	998 kg/m^3^	(Kumar et al., 2014)
	Specific heat capacity	
*cp*_*in*_	*c*_*p*_(*x*,*T*) (J/kgK)	Eq. (23) Current study
*cp*_*a*_	1000 J/kgK	(Kumar et al., 2014)
*cp*_*w*_	1000 J/kgK	(Kumar et al., 2014)
	Thermal conductivity	
*k*_*in*_	k(x,T) (W/mK)	Eq. (24) Current study
*k*_*a*_	0.0285 W/mK	Karim and Hawlader (2005)
*k*_*w*_	0.59 W/mK	(Cengel and Boles, 2002)
	Viscosity	
*μ*_*a*_	1.81e-5 kg/ms	(Cengel and Boles, 2002)
*μ*_*w*_	1.002e-3 kg/ms	(Kumar et al., 2014)

#### 2.2.2 Boundary condition

Based on the descriptions given in Section 2.2 and Fig. 1, the energy and mass boundary conditions are given by

(27) $$\text{Boundary conditions}\;\;=\left\{ \begin{array}{c} -k\frac{dT}{dn}=h_{T}(T_{s}-T_{a}),\;\text{Energy boundary equation}\\ -D\frac{dc}{dn}=h_{m}(C_{s}-C_{a}),\;\text{Mass boundary equation} \end{array}.\right.$$

The local heat ($h_{T}$) and mass ($h_{m}$) transfer coefficients along the interface between the channel flow and *injera* are defined by utilizing the following equations (Shen et al., 2020),

(28) $$h_{T}=\left\{ \begin{array}{c} 2\frac{k_{eff}}{L}\frac{0.3387Re^{1/2}Pr^{1/3}}{{\left(1+{\left(0.0468/Pr\right)}^{2/3}\right)}^{1/3}},\;Re\geq 5x10^{5}\\ 2\frac{k_{eff}}{L}Pr^{1/3}(0.037Re^{4/5}-871),\;Re\leq 5x10^{5} \end{array},\right.$$

(29) $$h_{m}=\frac{D_{air}}{L}(2+0.552R_{e}^{1/2}S_{c}^{1/3}),$$

where $k_{eff}$ is the effective thermal conductivity of *injera* (W/mK) and L is the characteristic length (cm). The Reynolds number (*Re*), Prandtl number (*Pr*) and Schmidt number (*Sc*) in Eqs. (28) and (29) can be expressed by Eqs. (30) to (32) respectively (Yu et al., 2020), where $\rho_{a}$ is the density of air, $\mu_{a}$ is the viscosity of air, $C_{pa}$ is the specific heat capacity of air, $D_{a}$ air diffusivity and $u_{a}$ is velocity of air.

(30) $$R_{e}=\frac{\rho_{a}u_{a}L}{\mu_{a}}$$

(31) $$P_{r}=\frac{C_{pa}\mu_{a}}{k_{a}}$$

(32) $$S_{c}=\frac{\mu_{a}}{\rho_{a}D_{a}}$$

## 3 Materials and methods

Based on its popularity among Ethiopian teff farmers and users, the qounchotef variety was selected and obtained from the Bahirdar Agricultural research Center and the *injera* sample was analysed according to the method of Gebremariam et al. (2014).

### 3.1 Chemical composition

The chemical composition of *injera* was determined according to well known tabulated methods. The moisture content was determined by the gravimetric method at 378.15 K (Horwitz et al., 2010; Baur and Ensminger, 1977), fat content was determined by the Soxhlet method (Kasiramar, 2019), ash content was determined by incineration in a muffle furnace at 823.15 K until there was no carbon left (Baur and Ensminger, 1977; Horwitz et al., 1970), the protein content was obtained by the Kjeldahl method (Bradstreet, 1954) and carbohydrates were obtained as the difference (Horwitz et al., 1970).

### 3.2 Experimental setup for drying

**Figure 2:**
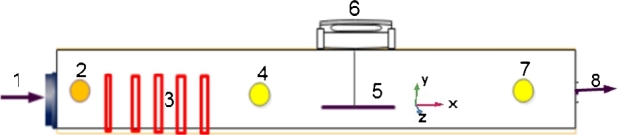
2D schematic diagram of the drying equipment.

Fig. 2 shows a schematic of the experimental set-up with a chamber size of 0.3×0.4×0.5 m. It was equipped with an electrical heater (3), air blower (1), air outlet chamber (8) and an electronic balance (6). A thermocouple, thermal anemometer and humidity transmitter (2,4,7) were used to measure the temperature, velocity and relative humidity. The air was supplied by an air blower with a damper used for adjusting the airflow rate. The air was heated by an electric heater and its velocity was measured by a thermal anemometer with an accuracy of ±0.01 ms^−1^ and its temperature by a K-type thermocouple with an accuracy of ±1°C. The values of air temperature (323.15 and 333.15 K) were fixed for the set point. The air humidity was determined by a psychrometer and it was 15.7 and 11% for the temperatures of 323.15 and 333.15 K respectively. For the experimental investigation, an *injera* sample of 3 cm thickness and 30 cm diameter was used for drying. The initial temperature of the sample was 298.15 K. The sample was put on a tray (5) made of a plastic net which was placed in front of a forced airflow system. It was used to reduce conduction heat transfer between try plate and sample. Each run included approximately 100 g of material. During the experiments, the weight of the sample was continuously measured until no noticeable difference was observed anymore.

### 3.3 Numerical approach

In this study, coupled heat and mass transfer in convective drying of *injera* was solved by the finite element method using commercial software (COMSOL Multiphysics, version 5.5). The choice for this finite element based software is due to the complex nature of the coupled heat and mass transfer models (Feyissa et al., 2013). The definitions of geometry, mesh size, material properties, boundary conditions and physical models were implemented in the package and both solving and post-processing of the data was done using the same software. The calculation was performed with laminar airflow through the *injera* sample. The Multifrontal Massively Parallel Sparse (MUMPS) direct solver was used to solve the governing equations. The simulation runs were carried out on a local computer, intel (R) Core (TM) i5 with 2.4 GHz processor speed, 8.00 GB installed RAM, operating under Windows 10 (64 bit).

### 3.4 Grid and time step independence analysis

**Figure 3:**
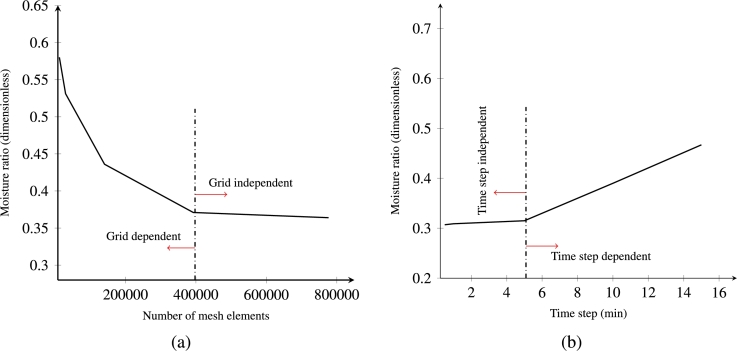
Computational independence analysis (a) grid independence (b) time step independence.

For credible simulation results, choosing an appropriate grid and time step size is important. The grid independence study for the moisture ratio (MR) was conducted from extremely course to fine mesh sizes and time steps ranged from 0.5 to 15 min as shown in Fig. 3. The moisture content was measured at the center of sample. It can be seen that the moisture ratio decreases up to a certain mesh size of about 400000 elements and that the solution becomes mesh independent when increasing the cell count further. Fig. 3b also shows the same mechanism for the time step: at low time step, the measured moisture ratio was almost constant up to a certain value (5 min) which shows the time step independency for time steps below 5 minutes.

### 3.5 Statistical tests and validation

Drying kinetics was modeled by means of three semi-empirical equations widely used in most food and biological materials, namely Henderson–Pabis in Eq. (34), two term model in Eq. (35) and Page in Eq. (36) (Omolola et al., 2014). All these models used the dimensionless moisture ratio (MR) as the dependent variable, defined as

(33) $$MR=\frac{M_{ava}-M_{e}}{M_{o}-M_{e}},$$

where $M_{e}$ is the equilibrium moisture content, $M_{ava}$ is the moisture content at time t and $M_{o}$ is the initial moisture content at t_*o*_.

Empirical correlations of the moisture ratio, involving only velocity and air temperature as a variable, are given as (Tunckal and Doymaz, 2020).

(34) $$MR=\alpha_{1}exp(-\alpha_{2}t),$$

(35) $$MR=\beta_{1}exp(-\beta_{2}t)+\beta_{3}exp(-\beta_{4}t),$$

(36) $$MR=exp(-\gamma_{4}t^{n}).$$

The correlation coefficient ($R^{2}$) and root mean square error (RMSE) were major criteria for selection of the best model equation to describe the drying curve. For best fit, $R^{2}$ (square of the Pearson correlation coefficient (r)) should be high and RMSE should be low (Cárcel et al., 2011; Demir et al., 2004; Ertekin and Yaldiz, 2004; Garau et al., 2006). In order to evaluate the goodness of fit of the simulation provided by the model, root means square error and Pearson correlation coefficient were provided in Eqs. (37) and (38) respectively. In the above equations, $y_{i}$ is the *ith* predicted moisture ratio, $x_{i}$ is the *ith* experimental moisture ratio, $\overset{¯}{y}$ is predicted sample means, $\overset{¯}{x}$ is experimental sample means and n is the number of observations.

(37) $$RMSE=\sqrt{\frac{1}{n}\sum_{i=1}^{n}{\left(x_{i}-y_{i}\right)}^{2}}$$

(38) $$r=\frac{\sum (x_{i}-\overset{¯}{x})(y_{i}-\overset{¯}{y})}{\sqrt{\sum {\left(x_{i}-\overset{¯}{x}\right)}^{2}{\left(y_{i}-\overset{¯}{y}\right)}^{2}}}$$

## 4 Results and discussion

### 4.1 Proximate composition of *injera*

The contents of carbohydrate, protein, crude fiber, ash and fat of dried *injera* were found to be 31.26±1.2 g/100 g, 4.85±0.1 g/100 g, 1.63±0.06 g/100 g, 1.83±0.1 g/100 g and 0.46 ±0.02 g/100 g respectively. The percentage deviation with previous reports for carbohydrate, protein, crude fiber, ash and fat contents of dried *injera* was 7.3, 4.1, 8.7, 8.5, 3.2 and 6.7% respectively, as it depends upon maturity, soil and the type of teff cereal (Haard, 1999). The moisture content of *injera* showed a value of 60.8±1.65 g/100 g which is in agreement with previous work, between the range of 60 to 65 g/100 g sample (Ashagrie and Abate, 2012).

### 4.2 Thermo physical properties

The thermophysical properties of *injera* as a function of composition and temperature are variable instead of constant. The density of *injera* was 1136.49 and 1135.41 kg/m^3^ at a temperature range of 323.15 K to 333.15 K respectively. This shows a reduction of density with increasing temperature due to the molecular kinetics (motion) of fluid, which increases the void space (Marcotte et al., 2008). The specific heat capacity of the *injera* sample was determined by the water content (60%) with showed values of 3676 to 3675.5 J/kgK at a temperature range from 323.15 K to 333.15 K. It did not have a significant variation when the temperature was increased (Shemishere et al., 2018). The thermal conductivity value ranges a maximum value of 0.3657 at a temperature of 313.15 K and a minimum value of 0.3434 W/m°C at a temperature of 333.15 K. An increasing temperature slightly decreases the thermal conductivity because of the inter molecular spacing is much larger and the motion of the molecules is more random in the fluid part and there is an increased lattice vibration in the solid content of *injera*. Therefore, the thermal conductivity decreases with increasing temperature (Telis-Romero et al., 1998).

### 4.3 Moisture ratio and temperature curves

**Figure 4:**
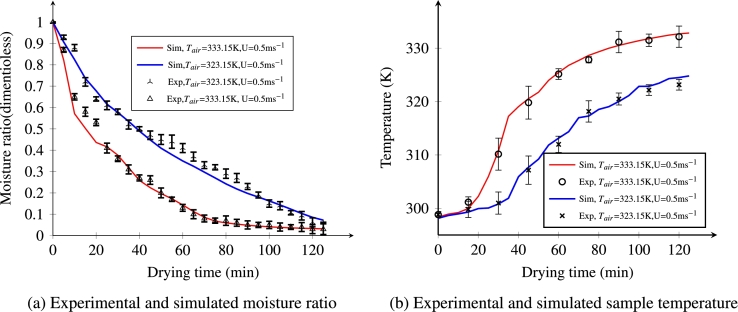
Experimental and simulated moisture ratio and temperature at constant air velocity (0.5 ms^−1^).

The results in Fig. 4a below clearly indicate the predicted moisture content of *injera* with the corresponding experimental data at an inlet air temperature of 323.15 and 333.15 K and an inlet velocity of 0.5 ms^−1^. The figure illustrates typical falling rate drying stages and there is a short constant drying stage for most food drying processes (Vásquez-Parra et al., 2013). In the early drying time, the curves were significantly reduced and termed as first stage failing as the drying was controlled by external conditions (the area exposed to dry air, temperature and velocity). In a later stage, the reduction was low and mainly controlled by internal mass transfer conditions (thickness and internal structure of the material), the so called second stage. As can be seen from the figure below, the simulated results agreed well with the experimental data. This is affirmed because the experimental data are bounded around the simulated solid line, indicating the suitability of the model for predicting the drying behavior of *injera*. An increasing drying temperature caused an important increase in the drying rate, thus the drying time was decreased. The time taken to reduce the moisture content of *injera* from the initial dimensionless moisture ratio of 1 (-) to a final 0.03(-) was 125 min at an air temperature of 333.15 K. However, it took about 150 min to reach the same dryness effect at 323.15 K. A similar result was observed in other works (Hussain and Dincer, 2003; Lemus-Mondaca et al., 2013; Villa-Corrales et al., 2010).

Fig. 4b shows the typical trends of the product temperature at hot-air temperatures (333.15 and 323.15 K) during drying of *injera*. From the figure, three different stages can be observed. In the early time, the product temperature was lower than the ambient air temperature. This could be explained by the evaporating cooling effect due to the rapid moisture flux on the surface during this period. It requires more energy for moisture evaporation and hence less heat received by the product initially. As the surface moisture was already removed from the sample by convective flow on the outside, water moves from the core towards the surface due to the low water potential there. The transport phenomena are controlled by internal flow conditions. Finally, equilibrium is reached with the drying air temperature remaining in a steady state. The temperature values obtained from the numerical simulation were in reasonable agreement with the measured temperature values obtained from the hot-air drying experiment ($R^{2}>0.95$). The reason for slight discrepancies might be attributed to the limited information or approximate representation of material properties for the *injera* as they change during the process. A similar observation was reported by Doymaz (2004).

### 4.4 Velocity distribution in the drying chamber

**Figure 5:**
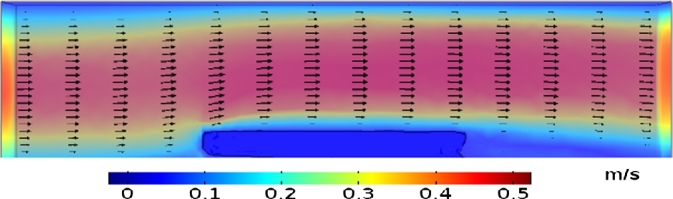
Velocity contour plot at inlet air velocity (0.5 ms^−1^).

Fig. 5 illustrates the fluid flow distribution in the drying chamber. The velocity values decrease when approaching the wall of the drying chamber due to the resistance between the wall and the fluid itself. Higher velocity values were observed in the center of the drying chamber. Also, the food acts as a bluff body to the flow creating a recirculation zone behind it. In this zone the velocities are low, hindering the removal of moist and heat transfer at the back (Khan et al., 2020; Pham et al., 2020).

### 4.5 Surface moisture distribution on the *injera*

**Figure 6:**
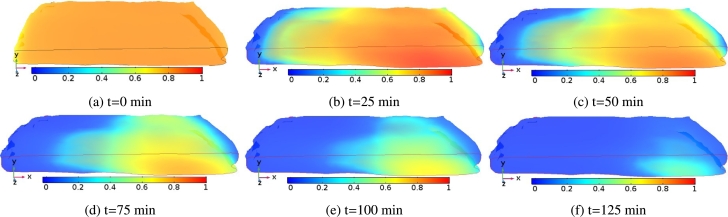
Schematic representation of surface moisture ratio distribution on the *injera* sample domain profile at an air temperature of 333.15 K and air velocity of 0.5 ms^−1^.

**Figure 7:**
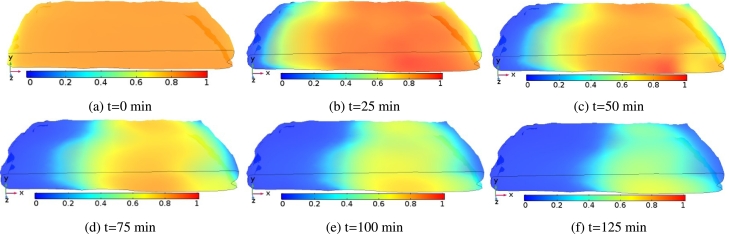
Schematic representation of the surface moisture ratio distribution on the *injera* sample domain profile at air temperature of 323.15 K and air velocity of 0.5 ms^−1^.

Fig. 6 shows the time evolution of moisture distribution of *injera* at different drying times. In this figure, the evolution of moisture in the sample was portrayed for an air temperature of 333.15 K at an air velocity of 0.5 ms^−1^. During the initial drying time (t=0 min), the dimensionless moisture distribution across the sample surface is virtually uniform as can be seen in Fig. 6a. As the drying proceeds, high surface moisture evaporation has occurred due to a moisture gradient between the *injera* and the surrounding air. In addition, the *injera* sample gained enough heat to remove moisture, creating a moisture difference between the surface and the inner core of the sample (Datta et al., 2007). This is attributed to the fact that a high amount of water evaporates from the inside of the *injera* to the surface and this water is removed by the surrounding air due to convective hot air currents during the first stage of the drying process Figs. 6b to 6e. Eventually, the rate of moisture transfer ceases due to internal mass transfer resistance that has come into play during high temperature drying because of bounded moisture in the sample. This results in lowering the rate of moisture migration to the surface despite the application of high temperatures and it took a great deal of time to reach the equilibrium moisture content, beyond which no further moisture removal occurred (MR=0.03), as shown in Fig. 6f. However, unlike at 333.15 K, there was more need of time and energy to remove the remaining moisture content from the sample at 323.15 K Figs. 7a to 7f. Similar phenomena were observed in most previous works (Aregawi et al., 2014a; Tzempelikos et al., 2015; Villa-Corrales et al., 2010).

### 4.6 Surface temperature distribution on the *injera*

**Figure 8:**
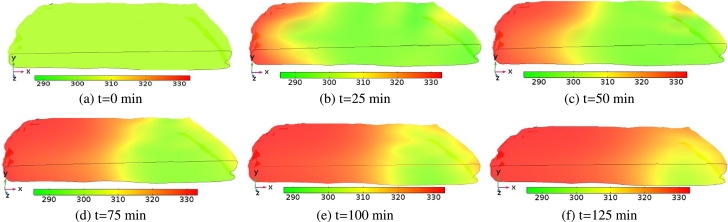
Schematic representation of surface temperature distribution profile on the *injera* sample domain at an air temperature of 333.15 K and air velocity of 0.5 ms^−1^.

**Figure 9:**
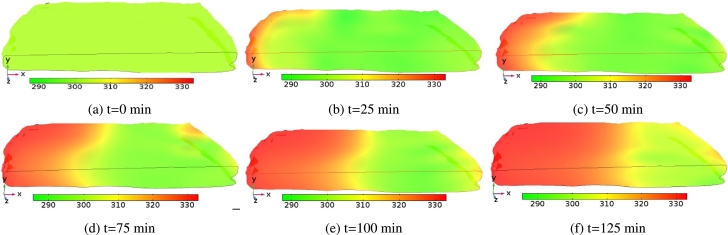
Schematic representation of surface temperature distribution profile on the *injera* sample domain at an air temperature of 323.15 K and air velocity of 0.5 ms^−1^.

Fig. 8 shows the temperature distributions of the *injera* at different drying times. In this figure, the evolution of temperature inside the sample was shown for an air temperature of 333.15 K and air velocity of 0.5 ms^−1^. At the initial drying time (t=0 min), the temperature profile at the sample surface is virtually uniform and in equilibrium with the ambient temperature, Fig. 8a. At the beginning of the drying process, moisture evaporation at the surface of the *injera* is dominant and heat removal by evaporation prevents heating up of the interior part of the sample, Figs. 8a to 8c. As the drying proceeds, the evaporation rate decreases and the core of the sample gradually warms up towards the drying air temperature, Figs. 8e and 8f. It was found that the temperature gradient at a drying temperature of 323.15 K that had been observed in Figs. 9a to 9f followed the same trend as in Figs. 8a to 8f except for the excessive time consumed to reach the equilibrium temperature. The same trends were observed by other works (Aregawi et al., 2014a; Villa-Corrales et al., 2010).

### 4.7 Effect of air velocity and temperature on the drying rate

**Figure 10:**
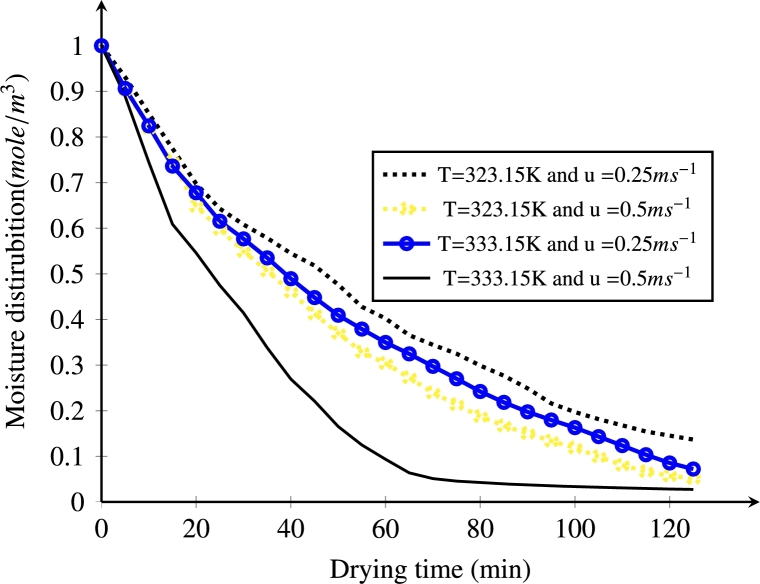
Effect of air velocity and temperature on moisture reduction rate.

ANOVA results showed that both temperature and velocity were significantly more interesting in moisture reduction when drying was conducted (p $<1.4\times 10^{-8}$). As it can be seen from the ANOVA results, the interaction effect between temperature and velocity also indicates a significance difference (p$<2.17\times 10^{-5}$). Fig. 10 depicts the changes in moisture content at different air velocities (u=0.25 and 0.5 ms^−1^) and drying air temperatures (323.15 and 333.15 K). For the first 20 min, moisture reduction was collapsed due to the dominant effect of external drying conditions. Higher air velocity and temperature induce a faster moisture transport rate. As the convective flow of moisture on the sample surface increases, the diffusion of liquid from the inner core of the *injera* to the surface followed by an evaporation towards the air domain increases. A similar expression was seen from Ateeque et al. (2014); Gulati and Datta (2013); Zhang and Datta (2004). As shown in the figure below, the effect of increasing temperature on the drying process is dominant over the increased velocity. From the figure it can be seen that drying rate is very high at the beginning. However, the surface gets dried quickly. Therefore, in a later stage, the velocity magnitude has a small effect on the evaporation because moisture levels on the surface are low. A similar behavior was observed by other studies (Pasban et al., 2017; Villa-Corrales et al., 2010).

### 4.8 Effective moisture diffusivity of the *injera*

Experimentally obtained effective moisture diffusivity Table 3, of the *injera* was increasing with rising of airflow rate and air temperature. The minimum value of moisture diffusivity was 6.29e-9 at an air temperature of 323.15 K and air velocity of 0.25 ms^−1^ and the maximum value was 1.29e-8 at an air temperature of 333.15 K and air velocity of 0.5 ms^−1^. Higher drying air temperature and air velocity resulted in the increment of the effective moisture diffusivity due to an increased moisture loss on the sample surface. A similar observation was reported in previous studies (Arslan and Özcan, 2010; Pasban et al., 2017)

**Table 3:** Average effective moisture diffusivity.

T (K)	u (ms^−1^)	*D*_*eff*_ (m^2^s^−1^)	*SD*
323.15	0.25	6.29e-9	±0.012
323.15	0.5	7.66e-9	±0.017
333.15	0.25	9.21e-9	±0.019
333.15	0.5	1.29e-8	±0.016

### 4.9 Moisture ratio distribution

**Figure 11:**
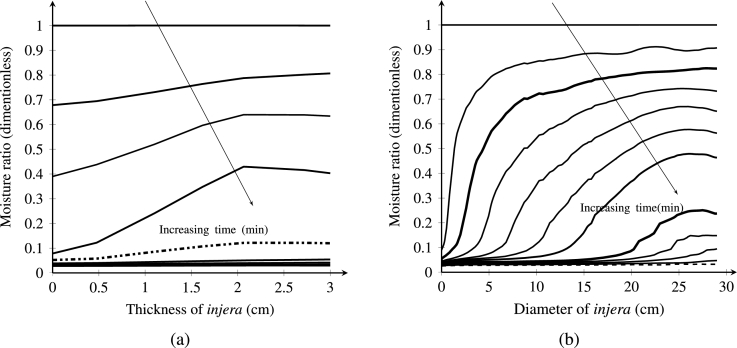
Moisture ratio distribution along the thickness and diameter (cm) of the *injera* at an air temperature of 333.15 K, relative humidity of 11% and velocity of 0.5 ms^−1^.

Fig. 11a illustrates the moisture distribution during the *injera* drying process at different positions (y-coordinate) and times (t=0,20,40,.. 120 min). The figure shows the moisture profile through the sample of the *injera* with increasing time. Moisture was decreased as the temperature rises with time. This decrease is the highest in the air inlet domain due to low air and surface temperature gradients and the gradients become higher as the thickness of the sample increases. Initially, the moisture content was uniformly distributed through the sample with a value of 1(-) and progressively reduces to 0.03 (-) with increasing time from 0 min to 125 min at a time interval of 20 min. As can be seen in the figure, moisture removal capacity was significant at the time range of 0 to 80 min. Because moisture gradient of the sample and air is high, this leads to dominant convective evaporation on the surface of the *injera*. After a time of 80 min, the reduction of moisture is dominated by diffusion.

Fig. 11b illustrates the moisture distribution in different x-coordinates and times. At the beginning, the moisture ratio of the *injera* is still high. The increased temperature over time reduces the moisture ratio at the surface due to the decreased water binding capacity at that location. The water transport from the inner part of the *injera* sample is large due to a high temperature gradient, creating a significant moisture gradient towards the center. This gradient decreases rapidly due to the low permeability of the still unheated *injera* towards the center (x > 5 mm). At a later time, the temperature gradient is drastically reduced and finally, at t=125 min, the temperature distribution became almost uniform which is called the steady state temperature. The same behavior was observed in the literature (Pasban et al., 2017; Tzempelikos et al., 2015; Villa-Corrales et al., 2010)

### 4.10 Temperature distribution

**Figure 12:**
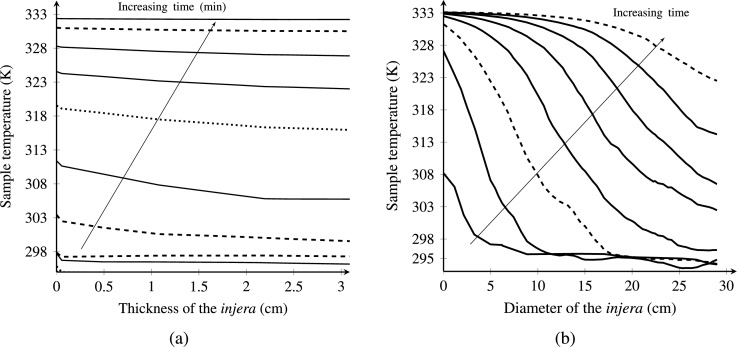
Temperature distribution along the thickness and diameter (cm) of the *injera* at an air temperature of 333.15 K, relative humidity of 11% and velocity of 0.5 ms^−1^.

Fig. 12a shows the temperature distribution during the *injera* drying process at different positions (y-coordinate) and times. In the early drying stage, the simulation shows a slight drop in product temperature, except for the top boundary at the *injera*-air interface which remains constant. The reason for this phenomenon is that the absorbed energy of heating was greater and acts as a sink for the incoming energy. As the drying time progresses, the temperature of the product also increases due to the removal of surface moisture from the sample and a rapid increment of product temperature. At later drying stages, its profile remained constant and becomes uniform, due to steady state conditions. A similar effect was reported in previous studies (Aregawi et al., 2014a; Datta, 2007b; Tzempelikos et al., 2015).

Fig. 12b shows the simulated temperature evolution along the diameter. It shows that the temperature is always higher on the surface of the *injera* compared to the inner part, despite the fact that air temperature and sample contact is higher at the surface. Initially the sample temperature is relatively low because of the convection and evaporation cooling which reduces the temperature at the surface. A similar pattern has been observed in the studies of Kumar et al. (2012); Shahari (2012). The temperature gradient is higher when x < 12 cm. The reason is that the hot air is staying most of its time at the back of the sample with increased heat transfer and yet facilitating the formation of vapor. As drying time increases, temperature of the product also increased significantly, which has remained constant right away throughout the coordinate of the sample.

### 4.11 Validation of moisture ratio and temperature curves

**Figure 13:**
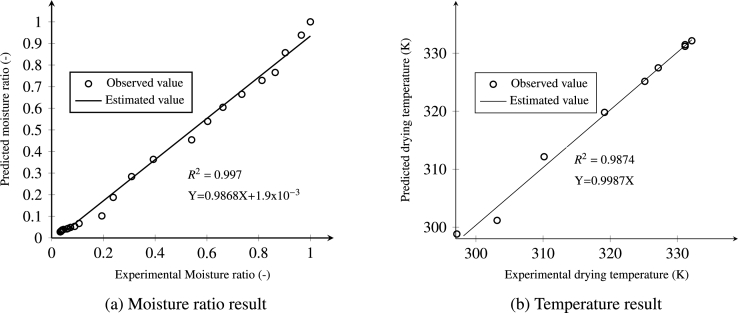
Simulated versus experimental moisture ratio and temperature of the *injera* at an air temperature of 333.15 K, relative humidity of 11% and velocity of 0.5 ms^−1^.

Fig. 13a illustrates the experimental and numerical moisture values at drying conditions of 333.15 K temperature, RH=11% and velocity of 0.5 ms^−1^. Both root mean square error (RMSE) and coefficient of determination ($R^{2}$) of moisture rate were 0.045 and 0.9874 respectively, which are indicators of how much the model approaches the experimental values. The model was reasonably acceptable. A similar observation was reported by (ElGamal et al., 2017; Zielinska and Markowski, 2010).

Fig. 13b shows the comparison of the measured value of temperature at the center of the food sample, which was recorded every 15 min during a 125 min total drying time, with the corresponding simulated result. The coefficient of determination $R^{2}$ is 0.9874 and root mean square error, RMSE=3.5 K. These figures indicate that the measured and simulated temperatures are in reasonable agreement, which in turn implies that the model is capable of predicting the temperature distribution at the center of the sample. These findings are in agreement with the result reported by (Doymaz, 2004; Zielinska and Markowski, 2010).

### 4.12 Comparison of the *injera* and other drying kinetics models

**Figure 14:**
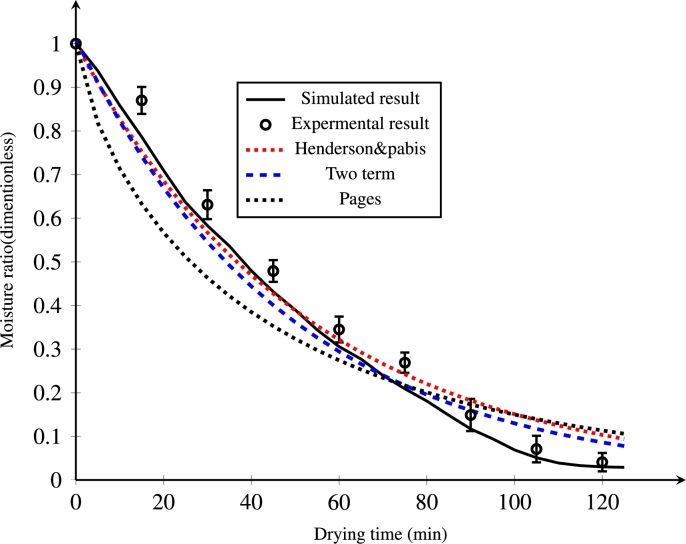
Comparison of simulated drying kinetics and well known mathematical models at an air temperature of 333.15 K and velocity of 0.5 ms^−1^.

Comparison of drying kinetics models (Henderson and Pabis, two-term, Pages) with the current *injera* drying process model is shown in Fig. 14. The drying models were fitted to the drying data and sorted in descending order of $R^{2}$ and ascending order of RMSE. The values of $R^{2}$ and RMSE of the drying models are shown in Table 4. From these results, the best statistical result was achieved with the Henderson and Pabis equation ($R^{2}=0.9952$ and RMSE = 0.04). For this reason, the close approximation by this model can be observed in the figure. Good agreement was observed at the beginning. Nevertheless, a significant deviation was seen after 80 min due to the nature of the moist object, which affects liquid diffusion transport towards the surface. In general, two terms and Pages also showed a good agreement between the mathematical model and the experimental moisture results as also confirmed by Aregawi et al. (2014b); Lemus-Mondaca et al. (2013); Tzempelikos et al. (2015). The comparison clearly indicates that the coupled heat and mass transfer numerical model of this study can give a better prediction of the drying characteristics of *injera*.

**Table 4:** Kinetics modeling of drying rate (MR) as a function of time (min).

Models	Constants	*R*^2^	*RMSE*
Henderson & Pabis	*α* = 1, *γ*_1_ = −0.0189	0.9952	0.04
Two term	*β*_1_ = 0.875, *β*_2_ = 0.135, *γ*_2_ = −0.021, *γ*_3_ = −0.0236	0.9916	0.047
Page	*γ*_4_ = −0.06, *n* = 0.745	0.9617	0.087

## 5 Conclusion

A coupled mathematical model of heat and mass transfer during drying was developed to study the drying process of *injera*. Moisture ratio and temperature distributions in the sample as a function of time were predicted. Based on the simulation results, the high value of the coefficient of determination (R>20.95 for both moisture and temperature) and low value of root mean square error (RMSE < 3.5 K for temperature and <0.05 for dimensionless moisture ratio) showed the good agreement between experiments and simulations. It can be concluded that temperature, velocity and their interaction has a significant effect on the moisture removal rate (p < 0.05). This model provides a better understanding of the heat and moisture transport phenomena inside the sample and the study could be safely used for further practical applications and serves as a theoretical basis for the analysis of food drying processes.

### Author Contribution Statement

Alamrew B. Solomon: Conceived and designed the experiments; Performed the experiments; Analyzed and interpreted the data; Contributed reagents, materials, analysis tools or data; Wrote the paper. Solomon W. Fanta: Conceived and designed the experiments; Analyzed and interpreted the data; Contributed reagents, materials, analysis tools or data; Wrote the paper. Mulugeta A. Delele; Maarten Vanierschot: Analyzed and interpreted the data; Wrote the paper.

### Funding Statement

This research did not receive any specific grant from funding agencies in the public, commercial, or not-for-profit sectors.

### Data Availability Statement

Data will be made available on request.

### 5.1 Declaration of Interests Statement

The authors declare no conflict of interest.

### Additional Information

No additional information is available for this paper.
